# Reduction of insulin resistance in 14–15-year-old students after the COVID-19 pandemic: a prospective study from Tsunan, Japan

**DOI:** 10.3389/fcdhc.2025.1687294

**Published:** 2026-01-07

**Authors:** Takayuki Ohno, Mizuki Ishiguro, Yuka Suganuma, Hironari Sano, Yusaku Hayashi, Rimei Nishimura

**Affiliations:** 1 The Jikei University School of Medicine, Tokyo, Japan; 2 Tsunan Town Hospital, Niigata, Japan

**Keywords:** COVID-19, HOMA-IR, insulin, insulin resistance, obesity

## Abstract

**Introduction:**

A significant increase in HOMA-IR values has been reported in children after the COVID-19 pandemic. This study aimed to investigate how the changes of HOMA-IR after COVID-19 was post-pandemic (2023–2024).

**Materials and methods:**

The study included 462 students aged 14–15 from Tsunan Town, Japan, who underwent health examinations between 2015 and 2024 (258 boys, 204 girls). The students’ HOMA-IR, BMI, and obesity levels were studied, and temporal changes were assessed using the Kruskal-Wallis test. IR was defined as HOMA-IR ≥2.5 and temporal changes were assessed using the chi-square test.

**Results:**

A significant change in the median HOMA-IR was observed over the 10-year period (p < 0.001). The proportion of IR was significantly higher in 2020, 2021, and 2022 (p < 0.001). Conversely, no significant differences were observed in the median BMI and obesity levels over the 10-year period (p = 0.18, p = 0.13). Significant correlations were observed between HOMA-IR and BMI as well as obesity levels throughout the entire observation period and from 2015 to 2019. However, no significant correlations were observed in the years 2020–2024.

**Discussion:**

The significant increase in HOMA-IR observed after 2020 significantly decreased to values similar to pre-COVID-19 levels by 2023. However, BMI and obesity levels showed no temporal changes. Our findings suggest that changes in lifestyle due to the COVID-19 pandemic during 2020–2022 may have influenced IR in 14–15-year-old students, irrespective of obesity status.

## Introduction

In Japan, annual school health checkups are mandated by law, during which anthropometric measurements, including height and weight, are routinely obtained. Nationwide school health data from 2012 to 2021 have demonstrated an increasing trend in childhood obesity among Japanese students (1). Obese children often present with hyperinsulinemia and insulin resistance (IR), and worsening IR may lead to the development of metabolic syndrome, thereby contributing to early atherosclerotic changes and elevating the risk of future cardiovascular disease (2). However, previous studies in Japan have reported no clear association between obesity and HOMA-IR (3).

Following the onset of the COVID-19 pandemic in 2020, reductions in outdoor physical activity and changes in dietary habits among children have been widely documented (4, 5). These lifestyle disruptions may have influenced metabolic health, particularly IR. Motivated by this context, we previously examined temporal changes in HOMA-IR, body mass index (BMI), and degree of obesity among 14–15-year-old students (third-year junior high school students in Japan) before and after the COVID-19 pandemic. In that analysis, we found that although BMI and degree of obesity did not change significantly over time, the proportion of students with elevated HOMA-IR increased markedly after the pandemic began (6).

Increased IR following the COVID-19 outbreak has also been reported in other populations (7). However, few studies have extended their follow-up through 2024, when daily life in Japan had largely returned to pre-pandemic conditions. To address this gap, the present study expanded our previous analysis by incorporating data up to 2024. Our aim was to determine whether BMI, degree of obesity, and HOMA-IR levels changed after the resolution of the COVID-19 pandemic, and to examine how the relationships among these variables evolved over time.

## Materials and methods

### Study population

This study was conducted at the sole junior high school in Tsunan Town, Niigata Prefecture, Japan. In each survey year, the analysis included all third-grade students (aged 14–15 years) who newly reached this grade level in that year, underwent the annual school health checkup, and provided informed consent—along with consent from their guardians. Thus, the study population consisted of different individuals each year. Sex was recorded based on school records. As the study targeted all third-grade junior high school students, no exclusion criteria were established.

### Study design

In Japan, routine school health checkups mandated by law include anthropometric measurements such as height and weight. In this study, the standard school health examination was supplemented with fasting blood tests, including fasting plasma glucose, serum insulin, and HbA1c. All blood samples were collected in the morning after an 8–12-hour fasting period. Insulin concentrations were measured using an automated analyzer manufactured by Kanto Chemical Co., Ltd. (Cias INSULIN II). The assay is based on a latex agglutination immunoturbidimetric method. Calibration was performed using the manufacturer’s insulin calibrators (six concentration levels), and quality control was conducted with Qualitrol INSULIN controls (two levels). The intra-assay coefficient of variation was approximately 4%.

HOMA-IR and the degree of obesity were calculated using the following formulas:

HOMA-IR = fasting insulin (μU/mL) × fasting glucose (mg/dL)/405;

degree of obesity (%) = [(actual weight (kg) – standard weight (kg)) × 100]/standard weight (kg).

Standard body weight was determined using Japanese reference equations: 0.832 × height (cm) – 83.695 for 14-year-old boys, 0.766 × height (cm) – 70.989 for 15-year-old boys, 0.594 × height (cm) – 43.264 for 14-year-old girls, and 0.560 × height (cm) – 37.002 for 15-year-old girls. (8).

BMI z-scores were calculated according to WHO recommendations. (9) Overweight was defined as a BMI z-score >1 and obesity as a BMI z-score >2, whereas underweight was defined as a BMI z-score < –1 and malnutrition as a BMI z-score < –2.

The primary outcome measures included longitudinal changes in HOMA-IR, BMI, and degree of obesity; temporal changes in the proportion of students with increased IR (defined as HOMA-IR ≥ 2.5); and temporal changes in the prevalence of overweight. Secondary outcome measures included correlations between HOMA-IR and BMI or degree of obesity evaluated across four time periods: the entire study period (2015–2024), the pre–COVID-19 period (2015–2019), the COVID-19 period (2020–2022), and the post–COVID-19 period (2023–2024). Analyses were conducted separately for boys and girls.

### Statistical analysis

All outcome measures were summarized as medians with interquartile ranges (25th–75th percentiles). Longitudinal changes in HOMA-IR, BMI, and degree of obesity were examined using the Kruskal–Wallis test for the entire cohort and for analyses stratified by sex. Pairwise comparisons were conducted using the Mann–Whitney U test. The frequencies of insulin resistance (IR), overweight, and underweight were compared using chi-square tests with residual analysis, stratified by sex.

Correlations between HOMA-IR and either BMI or degree of obesity were assessed using Spearman’s rank correlation coefficients. These analyses were performed for the overall cohort, stratified by sex, and across three time periods: the pre–COVID-19 period (2015–2019), the COVID-19 period (2020–2022), and the post–COVID-19 period (2023–2024).

To further evaluate associations, multiple linear regression analysis was performed with HOMA-IR as the dependent variable and survey year, sex, and BMI z-score as explanatory variables. Logistic regression analysis was conducted with the presence of IR as the dependent variable and survey year as the primary independent variable, adjusting for sex and BMI z-score. Additionally, a linear mixed-effects model was fitted with HOMA-IR as the dependent variable and sex, BMI category, survey year category, and their interaction terms as explanatory variables. BMI was dichotomized based on the presence or absence of obesity, and survey year was categorized into two groups according to whether measurements were obtained during the COVID-19 pandemic period.

Statistical significance was defined as a two-sided p value <0.05. All analyses were performed using SPSS version 29.0 (SPSS Inc., Chicago, IL, USA; www.spss.com).

The study was approved by the Ethics Committee of Jikei University School of Medicine (20-010 5200), conformed to the principles of the Declaration of Helsinki (as revised in 2000), and written informed consent was obtained from all participants and their guardians prior to study enrollment.

## Results

A total of 462 participants (258 boys and 204 girls) were included. The median height (25th–75th percentile) was 161.3 (156.1–167.0) cm, median weight was 51.4 (46.8–57.9) kg, median BMI was 19.8 (18.3–21.5) kg/m², median BMI z-score was 0.29 (–0.26–0.95), median HOMA-IR was 2.0 (1.5–2.7), and median degree of obesity was –1.0 (–7.7 to 8.2)% (**Table 1**).

**Table 1:** Demographic data.

	Overall	Boys	Girls	*P value* ^†^
number	462	258	204	–
Hight (cm)	161.3 (156.1–167.0)	166.0 (161.0–169.2)	157.3 (153.1–160.4)	<0.001***
Weight (kg)	51.4 (46.8–57.9)	52.8 (47.6–59.2)	49.6 (46.1–55.0)	<0.001***
BMI (kg/m^2^)	19.8 (18.3–21.5)	19.2 (17.8–21.3)	20.4 (18.8–22.1)	<0.001***
BMI z-score	0.29 (-0.26–0.95)	0.90 (-0.46–0.87)	0.52 (-0.07–1.18)	<0.001***
Degree of Obesity (%)	−1.0 (-7.7–8.2)	−1.7 (-8.5–8.2)	0.6 (-7.1–9.3)	0.151
FPG (mg/dL)	94.0 (90.0–98.0)	95.0 (91.0–100.0)	92.0 (89.0–96.0)	<0.001***
Insulin (μU/mL)	8.6 (6.5–11.8)	7.9 (6.1–10.8)	9.6 (7.3–12.8)	<0.001***
HOMA-IR	2.0 (1.5–2.7)	1.9 (1.4–2.6)	2.2 (1.6–2.9)	<0.001***
HbA1c (%)	5.5 (5.3–5.6)	5.5 (5.3–5.6)	5.5 (5.3–5.7)	0.245

Data are represented as median (25–75 percentiles [interquartile range]).

BMI, body mass index; FPG, fasting plasma glucose; HOMA-IR, Homeostasis model assessment of insulin resistance.

†Mann-Whitney test.

***P < 0.001.

HOMA-IR demonstrated significant longitudinal changes across the 10-year period from 2015 to 2024 (p < 0.001) (**Table 2**). Sex-stratified analyses also showed significant changes in both boys (p < 0.001) and girls (p = 0.001).

**Table 2:** Time trends of HOMA-IR, BMI and degree of obesity during 2015–2024.

		2015	2016	2017	2018	2019	2020	2021	2022	2023	2024	*P value*
overall n=462	n	60	62	57	47	56	47	37	39	25	32	–
	IR (%)	20(10-30)	19(9-29)	18(8-28)	31(18-45)	38(25-52)	61(47-75)	61(44-77)	74(60-88)	16(2-30)	3(0-9)	<0.001^††^***
	HOMA-IR	1.8 (1.2–2.3)	1.8 (1.5–2.3)	1.6 (1.3–2.2)	2.0 (1.5–2.6)	2.0 (1.4–3.1)	2.9 (2.0–3.2)	2.7 (2.2–3.0)	2.9 (2.4–4.0)	2.0 (1.5–2.3)	1.5 (1.3–1.7)	<0.001^†^***
	BMI (kg/m^2^)	19.8 (18.7–22.1)	19.7 (17.7–21.7)	19.6 (18.3–21.2)	19.7 (18.0–21.1)	20.4 (19.0–21.9)	18.7 (17.4–20.6)	19.7 (18.3–21.6)	19.9 (18.5–21.4)	20.1 (18.3–22.4)	20.2 (18.7–21.4)	0.18^†^
	Degree of obesity (%)	−0.5 (−7.5–11.0)	−0.9 (−7.5–9.2)	−0.8 (−7.8–7.7)	−1.9 (−9.6–5.0)	1.2 (−5.7–9.9)	−5.6 (−12.4–2.4)	−3.1 (−7.7–10.4)	1.9 (−6.1–8.0)	1.3 (−8.4–11.8)	2.4 (−5.1–8.6)	0.13^†^
boys n=258	n	37	41	40	12	32	24	15	18	17	22	–
	IR (%)	19(6-32)	19(2-24)	13(2-23)	33(7-60)	33(17-50)	54(34-74)	69(44-94)	82(64-100)	6(0-17)	5(0-13)	<0.001^††^***
	HOMA-IR	1.7 (1.3–2.4)	1.8 (1.5–2.1)	1.5 (1.2–2.0)	2.2 (1.6–2.9)	1.9 (1.3–3.0)	2.6 (1.9–3.3)	2.7 (2.2–2.8)	3.0 (2.5–3.8)	1.9 (1.5–2.3)	1.5 (1.3–1.7)	<0.001^†^***
	BMI (kg/m^2^)	19.8 (17.9–22.1)	19.7 (17.7–22.2)	19.3 (17.9–20.9)	17.5 (16.9–18.6)	19.3 (18.1–21.4)	18.9 (17.5–20.1)	18.4 (17.6–21.6)	18.6 (17.6–19.7)	20.1 (18.2–23.5)	20.1 (18.7–21.4)	0.49^†^
	Degree of obesity (%)	0.9 (−8.2–12.3)	−0.8 (−7.0–11.4)	−1.6 (−8.4–5.7)	−10.0 (−14.5–−5.1)	−2.6 (−6.2–7.8)	−4.0 (−10.6–3.5)	−5.0 (−9.8–13.4)	−3.8 (−12.1–4.6)	1.3 (−8.6–20.8)	3.6 (−4.3–9.2)	0.46^†^
girls n=204	n	23	21	17	35	24	23	22	21	8	10	–
	IR (%)	22(5-39)	29(9-48)	31(9-54)	30(15-46)	45(25-66)	68(49-88)	55(33-77)	67(47-87)	38(4-71)	0(0)	<0.01^††^**
	HOMA-IR	1.9 (1.2–2.2)	1.9 (1.6–2.5)	2.0 (1.5–2.6)	2.0 (1.5–2.6)	2.1 (1.6–4.4)	3.0 (2.2–3.2)	2.6 (2.2–3.1)	2.9 (2.3–4.1)	2.2 (1.6–3.2)	1.4 (1.3–1.7)	<0.001^†^***
	BMI (kg/m^2^)	19.9 (19.0–22.2)	19.9 (18.4–21.3)	21.1 (19.2–23.2)	20.1 (18.3–21.4)	21.5 (20.0–25.3)	18.5 (17.2–20.8)	19.7 (18.9–22.0)	20.9 (20.0–23.2)	20.3 (18.6–21.3)	20.5 (18.6–21.6)	0.006^†^**
	Degree of obesity (%)	−2.1 (−6.0–9.3)	−2.0 (−9.8–4.9)	4.4 (−5.1–16.0)	−0.6 (−6.4–6.1)	6.6 (−1.5–25.5)	−9.3 (−15.1–2.4)	−3.0 (−6.7–8.6)	2.6 (0.3–15.2)	0.1 (−8.2–5.2)	1.0 (−8.3–6.0)	0.006^†^**

Data are represented as median (25–75 percentiles [interquartile range]).

IR indicates the prevalence and 95% confidence interval.

HOMA-IR, Homeostasis model assessment of insulin resistance; BMI, body mass index.

†Kruskal-Wallis test. ††Chi-square test. *P < 0.05. **P < 0.01. ***P < 0.001.

**Figure 1:**
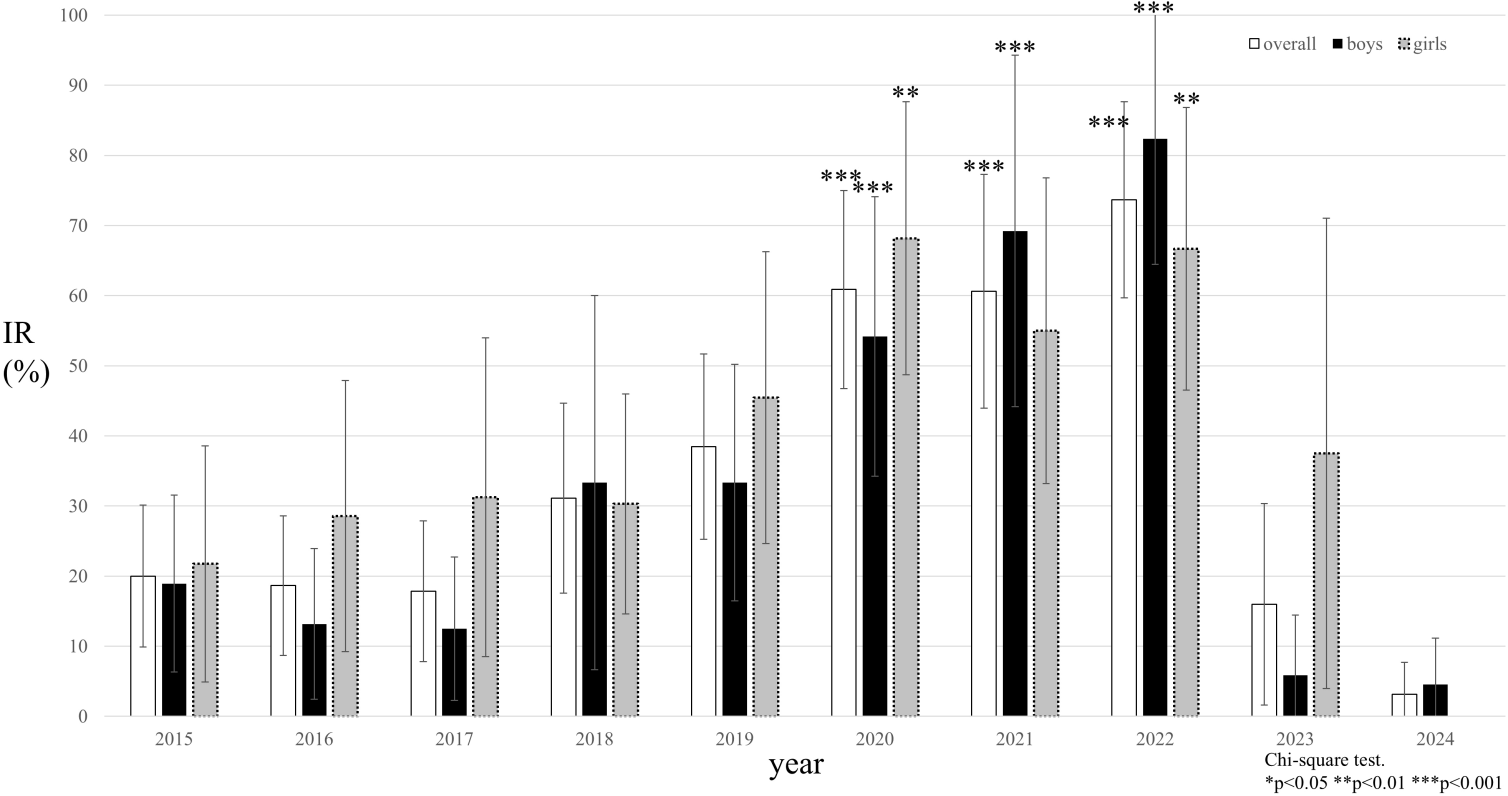
Changes in the proportion of study participants with HOMA-IR values of ≥2.5 (IR) over time. The white/black/gray bar graph illustrates the proportion of IR in overall/boys/girls. The sample sizes for each year were 60 in 2015, 62 in 2017, 57 in 2018, 47 in 2018, 56 in 2019, 47 in 2020, 37 in 2021, 39 in 2022, 25 in 2023 and 32 in 2024. Chi-square test with residual analysis. *p<0.05. ** p<0.01. ***p<0.001.

The proportion of students with increased IR was significantly higher during the COVID-19 years—2020 (60.9%), 2021 (60.6%), and 2022 (73.7%) (p < 0.001) (**Figure 1**). Sex-specific analyses showed significant increases in boys from 2020 to 2022 (p < 0.001) and in girls in 2020 and 2022 (p = 0.007).

In contrast to HOMA-IR, BMI did not exhibit significant longitudinal changes from 2015 to 2024 (p = 0.18). Sex-stratified analyses revealed significant differences across years only in girls (boys: p = 0.49; girls: p = 0.006), although no consistent temporal trend was observed, as seen with HOMA-IR. Similarly, the degree of obesity showed no significant temporal changes overall (p = 0.13). Sex-specific analyses identified significant differences across years only in girls (boys: p = 0.46; girls: p = 0.004), but again, no clear temporal pattern emerged.

**Figure 2:**
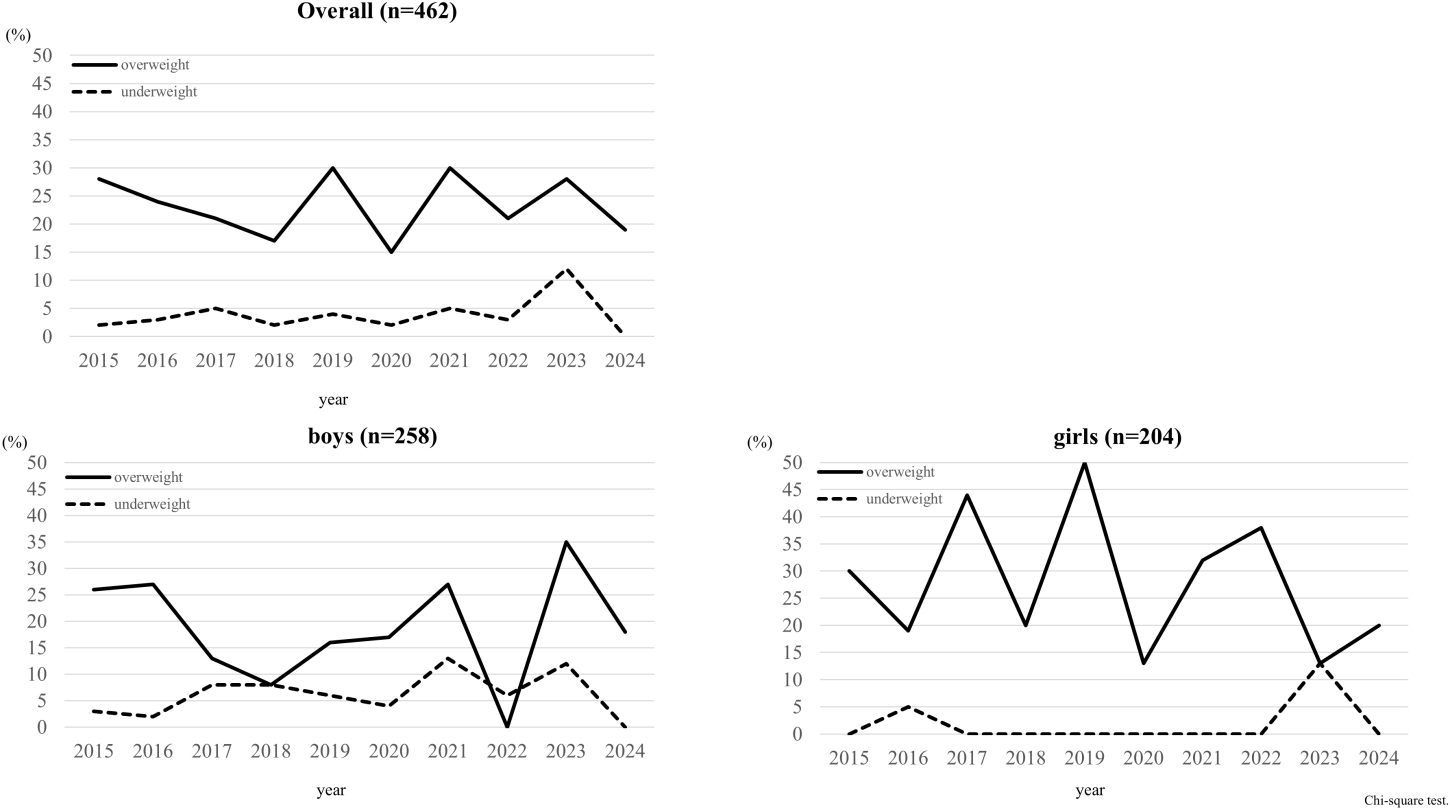
Changes in the proportions of overweight and underweight participants over time. Dot line illustrates the proportion of students defined as BMI z-score <1. Solid line illustrates the proportion of students defined as BMI z-score >1. The sample sizes for each year were 60 in 2015, 62 in 2017, 57 in 2018, 47 in 2018, 56 in 2019, 47 in 2020, 37 in 2021, 39 in 2022, 25 in 2023 and 32 in 2024. Chi-square test with residual analysis.

No significant temporal changes were observed in the prevalence of overweight or underweight in the overall cohort (p = 0.656 and p = 0.469, respectively), nor in analyses stratified by sex (overweight—boys: p = 0.194, girls: p = 0.103; underweight—boys: p = 0.723, girls: p = 0.074) (**Figure 2**). Even when stratified by overweight status, HOMA-IR showed significant temporal changes, whereas neither BMI nor degree of obesity demonstrated such changes (**Supplementary Table S2**).

**Figure 3:**
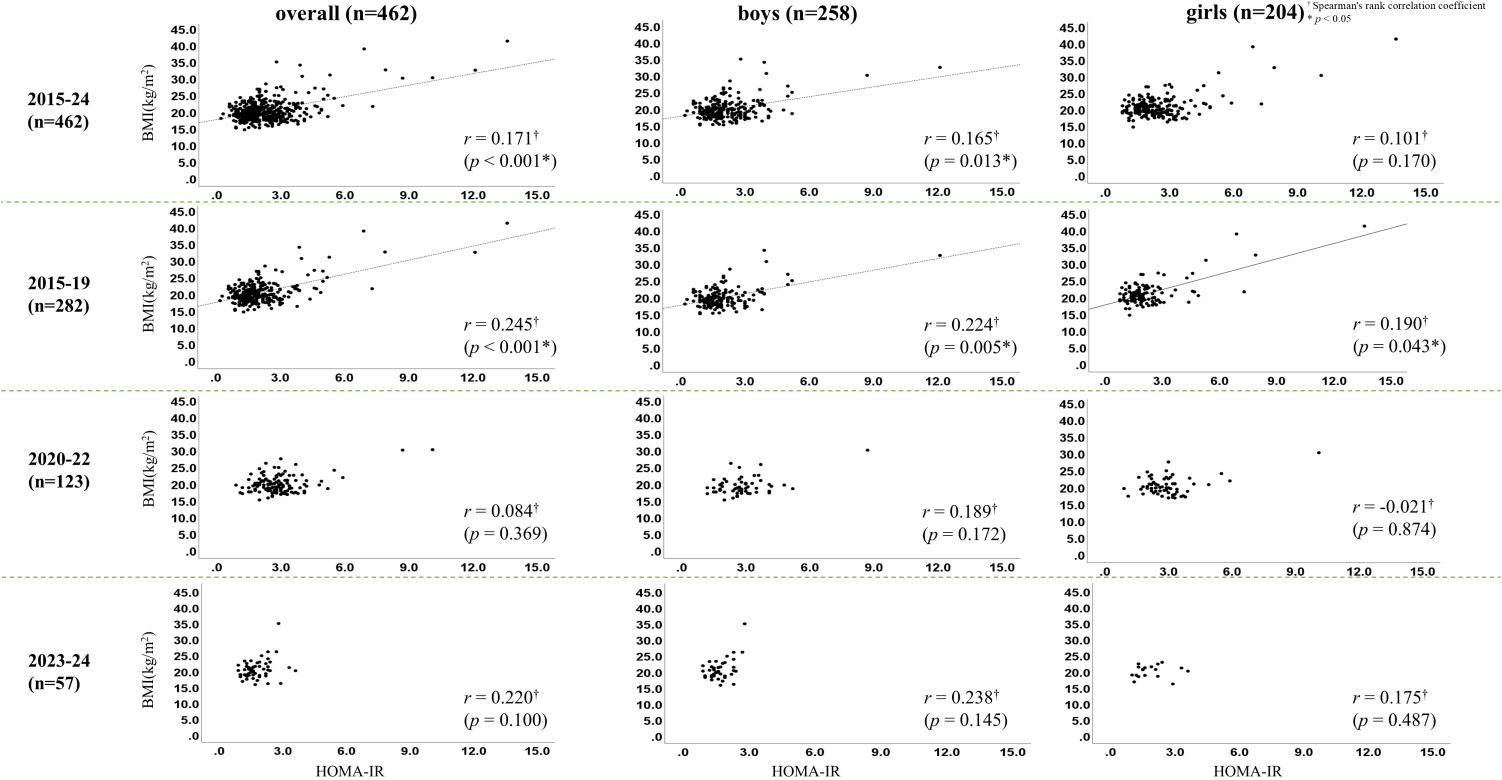
Correlations between homeostasis model assessment of insulin resistance (HOMA-IR) and body mass index (BMI) before (2015-2019) and after (2020-2022) the outbreak of the COVID-19 pandemic and after (2023–2024) the containment. ^†^Spearman’s rank correlation coefficient. *p < 0.05.

**Figure 4:**
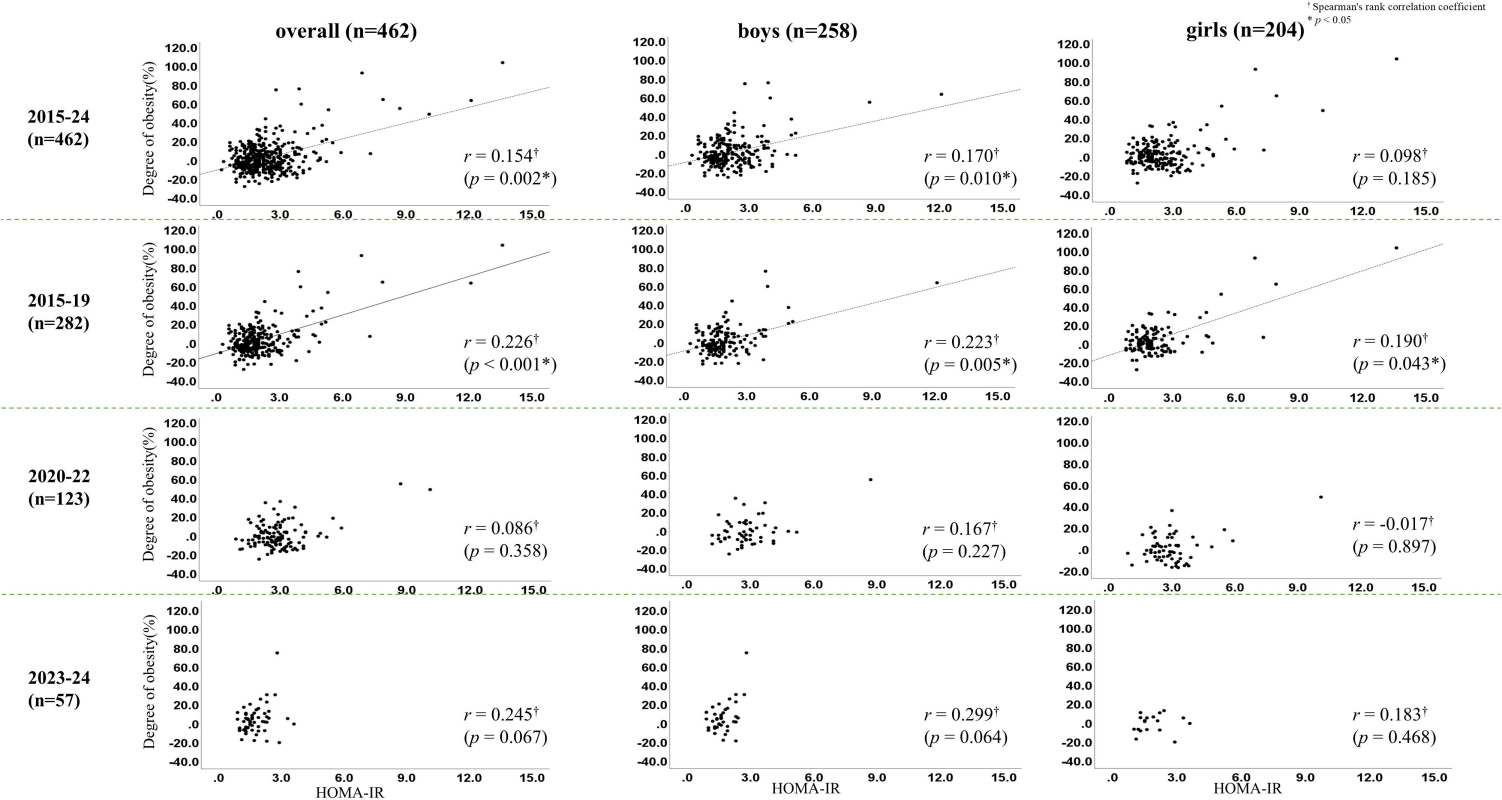
Correlations between homeostasis model assessment of insulin resistance (HOMA-IR), and degree of obesity before (2015-2019) and after (2020-2022) the outbreak of the COVID-19 pandemic and after (2023–2024) the containment. ^†^Spearman’s rank correlation coefficient. *p < 0.05.

In the overall analysis, significant correlations were observed between HOMA-IR and both BMI (r = 0.171, p < 0.001) and degree of obesity (r = 0.154, p = 0.002). These associations were particularly evident during the pre–COVID-19 period (2015–2019) (BMI: r = 0.245, p < 0.001; obesity degree: r = 0.226, p < 0.001). During the COVID-19 period (2020–2022), however, no significant correlations were detected (BMI: r = 0.084, p = 0.369; obesity degree: r = 0.086, p = 0.358). This absence of association persisted in 2023–2024 (BMI: r = 0.220, p = 0.100; obesity degree: r = 0.245, p = 0.067) (**Figures 3**, **4**). Sex-specific analyses showed significant correlations only in boys (BMI: r = 0.165, p = 0.013; obesity degree: r = 0.170, p = 0.010), whereas no significant associations were observed in girls.

In the multiple linear regression analysis, survey year (β = 0.055, 95% CI: 0.016–0.095, p = 0.006) and BMI z-score (β = 0.535, 95% CI: 0.445–0.626, p < 0.001) were significantly associated with HOMA-IR, whereas age was not (p = 0.127). The model explained 24.5% of the variance (R² = 0.245).

In the logistic regression analysis, the years 2020, 2021, and 2022 were significantly associated with increased IR prevalence (2020: OR = 8.21; 2021: OR = 7.48; 2022: OR = 13.22; all p < 0.001). Sex was not associated with IR prevalence (p = 0.492), whereas BMI z-score showed a strong association (OR = 1.46, 95% CI: 1.21–1.76, p < 0.001).

In the linear mixed-effects model, sex (β = –0.29, p = 0.011), BMI category (β = 1.64, p < 0.001), and survey year category (β = 0.86, p < 0.001) were significantly associated with HOMA-IR. However, the interaction between BMI category and survey year category was not significant (β = 0.13, p = 0.78).

After applying the Benjamini–Hochberg procedure to control the FDR at 5% across 45 Kruskal–Wallis comparisons, 26 comparisons remained significant (q ≤ 0.05). The significant q-values ranged from 0.0026 to 0.0225 (**Supplementary Table S3**).

## Discussion

This study is the first to demonstrate a significant increase in the prevalence of insulin resistance (IR) among Japanese students aged 14–15 years during the COVID-19 pandemic (2020–2022), followed by a marked decline in 2023. Furthermore, we identified a novel shift in the correlation patterns between HOMA-IR and both BMI and obesity levels after 2020. Notably, these correlations remained significant among boys but disappeared among girls throughout the study period.

The pronounced rise and subsequent decline in the prevalence of increased IR aligned closely with the timeline of the COVID-19 pandemic, suggesting a potential association between the pandemic-related lifestyle changes and increased IR. A nationwide physical fitness survey of more than 16 million Japanese children aged 10–11 and 13–14 years reported significant reductions in 20-meter shuttle run performance and abdominal muscle strength after the onset of the pandemic compared with pre-pandemic levels. (10) Consistent with these findings, students in our study experienced temporary school closures and reduced extracurricular activity opportunities, which likely contributed to decreased physical activity. Reduced physical activity has been linked to inflammatory cytokine elevation and impaired insulin sensitivity. (11).

Changes in dietary habits during the pandemic may also have contributed. In a study of 671 Japanese children aged 10–14 years, frequent consumption of instant foods was associated with poorer nutritional balance and a higher prevalence of nutrient deficiencies. (12) Another study involving 1,111 children aged 10–14 years reported a significant decline in the proportion of children consuming balanced diets after the declaration of the 2020 state of emergency. (13) Increased caloric intake and higher consumption of total fat and saturated fatty acids are known contributors to IR and obesity-related inflammation. (14, 15) Similar dietary changes may have occurred in our cohort.

Although post-pandemic lifestyle changes have been less extensively studied, a large-scale survey of 4,084 Japanese children aged 8–15 years found reduced intake of confectionery and sugar-sweetened beverages after school reopening compared with during school closures. (16) Such behavioral improvements may have contributed to the observed decline in IR in 2023.

A cutoff value of HOMA-IR ≥3.22, validated against the hyperglycemic clamp method, has been proposed for adolescents. (17) Using this cutoff, the prevalence of elevated IR was significantly higher in both 2020 (23.9%) and 2022 (36.9%). Sensitivity analysis using the weighted 90th percentile (HOMA-IR = 3.6) also revealed a significant increase in IR in 2022 in the overall cohort and among girls. Sex-specific analyses confirmed significant increases in boys in 2020 and 2022 and in girls in 2022. Although the prevalence did not significantly increase in 2021 when using the lower cutoff of ≥2.5, the overall trend—elevations in 2020 and 2022 followed by improvement in 2023—remained consistent across all thresholds.

Despite the significant temporal changes in IR, BMI and obesity levels showed no meaningful longitudinal change from 2015 to 2024. Although girls exhibited significant differences across years, no consistent upward or downward trends were observed. Similarly, analyses stratified by overweight status revealed no temporal change in BMI or degree of obesity, regardless of overweight classification.

These findings are consistent with reports that, in children and adolescents, IR may worsen even when BMI remains stable. A study in Japanese children aged 9–15 years found increased body fat percentage after the pandemic despite unchanged BMI. (18) Another study among children aged 10–12 years demonstrated reduced grip strength and two-step test performance post-pandemic (19). Reduced physical activity can increase body fat and decrease fat-free mass without substantially altering total body weight. This mechanism provides a plausible explanation for why IR increased while BMI did not.

Indirect evidence during the pandemic also supports this interpretation: suspension of club activities, increased parental car transportation instead of walking, and anecdotal reports of increased consumption of snacks such as juice and ice cream likely contributed to reduced physical activity and poorer dietary habits. Although direct measurements were not available, these factors may collectively explain the divergence between IR and BMI trajectories.

Before the pandemic, significant correlations between HOMA-IR and BMI, as well as between HOMA-IR and degree of obesity, were evident in both boys and girls. However, these correlations disappeared after 2020. This suggests that pandemic-related behavioral changes may have altered the relationship between adiposity and insulin sensitivity. Interestingly, correlations persisted among boys but not among girls, highlighting potential sex-specific responses that warrant further investigation. Although a re-establishment of pre-pandemic correlation patterns might have been expected in 2023–2024, this did not occur, potentially due to the limited sample size available for the post-pandemic years.

We hypothesize that pandemic-related lockdowns and restricted mobility contributed to the observed rise in HOMA-IR, possibly by increasing sedentary time and altering dietary habits. However, given the study’s observational design, this remains a testable hypothesis and cannot be established as a causal link.

### Limitations

This study has several limitations. First, its cross-sectional design at each survey year limits the ability to infer future cardiometabolic risk or establish temporal causality. Second, dietary intake and physical activity were not assessed or quantified, preventing accurate evaluation of their contributions to the observed findings. Information on Tanner stage was also unavailable, which may have influenced the interpretation of insulin resistance profiles in adolescents.

Third, the relatively small sample size highlights the need for larger studies involving more diverse populations. In addition, the number of participants varied across survey years, and the sample size was particularly limited in the post–COVID-19 period, which may have affected the stability and interpretation of temporal trends. Because the study was conducted within a single geographic region, the generalizability of our findings may be limited.

Finally, precise measurements of body composition were not obtained. Beginning in 2026, however, we plan to incorporate bioelectrical impedance analysis to collect more detailed body composition data and enhance the accuracy of future assessments.

## Conclusion

This study demonstrated that the prevalence of increased IR rose markedly among Japanese students aged 14–15 years during the COVID-19 pandemic period (2020–2022), before subsequently declining in the post-pandemic years (2023–2024). The findings underscore a critical aspect of pediatric health, indicating that alterations in lifestyle habits induced by the COVID-19 pandemic led to a substantial rise in HOMA-IR, even in individuals with normal BMI and obesity levels.

## Data Availability

The raw data supporting the conclusions of this article will be made available by the authors, without undue reservation.
