# Supplementary material for: Reduction of insulin resistance in 14–15-year-old students after the COVID-19 pandemic: a prospective study from Tsunan, Japan
Source: Front Clin Diabetes Healthc. 2026 Jan 7;6:1687294. doi: 10.3389/fcdhc.2025.1687294 (PMC12819293; doi:10.3389/fcdhc.2025.1687294)
Supplement: Supplementary file 1 [file SupplementaryFile1.docx]

**Table S1 | Demographic data by BMI z-score**

|  | overall | obesity (+) | overweight (+) | normal | underweight (+) | *P value*^†^ |
| --- | --- | --- | --- | --- | --- | --- |
| number | 462 | 37 | 70 | 335 | 16 | – |
| Hight (cm) | 161.3  (156.1–167.0) | 162.2  (156.9–168.2) | 160.7  (155.9–166.6) | 161.1  (156.0–166.9) | 166.4  (158.5–172.1) | 0.248 |
| Weight (kg) | 51.4 (46.8–57.9) | 73.0 (66.3–79.4) | 58.3 (54.6–62.6) | 49.2 (46.1–54.0) | 42.9 (40.6–46.7) | <0.001*** |
| BMI (kg/m^2^) | 19.8 (18.3–21.5) | 26.7 (25.3–30.4) | 22.6 (22.2–23.2) | 19.1 (18.0–20.3) | 15.7 (15.2–16.1) | <0.001*** |
| BMI z-score | 0.29 (-0.26–0.95) | 3.0 (2.4–4.4) | 1.40 (1.22–1.61) | 0.54 (-0.38–0.49) | -1.3 (-1.4–-1.1) | <0.001*** |
| Degree of Obesity (%) | −1.0 (-7.7–8.2) | 33.1 (27.6–53.5) | 13.6 (10.4–16.0) | −3.6 (–8.5–1.7) | -20.8 (-23.5–-19.0) | <0.001*** |
| FPG (mg/dL) | 94.0 (90.0–98.0) | 97.0 (89.0–101.0) | 93.5 (88.8–98.3) | 93.0 (90.0–98.0) | 94.5 (87.3–101.0) | 0.243 |
| Insulin (μU/mL) | 8.6 (6.5–11.8) | 12.8 (9.3–19.7) | 9.0 (6.6–11.9) | 8.2 (6.3–11.4) | 8.8 (6.8–11.0) | <0.001*** |
| HOMA-IR | 2.0 (1.5–2.7) | 2.9 (2.0–5.1) | 2.1 (1.6–2.8) | 1.9 (1.4–2.6) | 2.0 (1.5–2.6) | <0.001*** |
| HbA1c (%) | 5.5 (5.3–5.6) | 5.4 (5.2–5.7) | 5.5 (5.3–5.6) | 5.5 (5.3–5.6) | 5.5 (5.3-5.8) | 0.708 |

Data are represented as median (25–75 percentiles [interquartile range]).

BMI; body mass index, FPG; fasting plasma glucose, HOMA-IR; Homeostasis model assessment of insulin resistance.

†Kruskal-Wallis test.

***P < 0.001.

**Table S2 | Time trends of HOMA-IR, BMI and degree of obesity by BMI z-score during 2015–2024**

|  | | year | | | | | | | | | | *P value* |
| --- | --- | --- | --- | --- | --- | --- | --- | --- | --- | --- | --- | --- |
|  |  | 2015 | 2016 | 2017 | 2018 | 2019 | 2020 | 2021 | 2022 | 2023 | 2024 |  |
| overall  n=462 | n | 60 | 62 | 57 | 47 | 56 | 47 | 37 | 39 | 25 | 32 | - |
|  | IR (%) | 20 (10-30) | 19 (9-29) | 18 (8-28) | 31 (18-45) | 38 (25-52) | 61 (47-75) | 61 (44-77) | 74 (60-88) | 16 (2-30) | 3 (0-9) | <0.001^††^*** |
|  | HOMA-IR | 1.8  (1.2–2.3) | 1.8  (1.5–2.3) | 1.6  (1.3–2.2) | 2.0  (1.5–2.6) | 2.0  (1.4–3.1) | 2.9  (2.0–3.2) | 2.7  (2.2–3.0) | 2.9  (2.4–4.0) | 2.0  (1.5–2.3) | 1.5  (1.3–1.7) | <0.001^†^*** |
|  | BMI (kg/m^2^) | 19.8  (18.7–22.1) | 19.7  (17.7–21.7) | 19.6  (18.3–21.2) | 19.7  (18.0–21.1) | 20.4  (19.0–21.9) | 18.7  (17.4–20.6) | 19.7  (18.3–21.6) | 19.9  (18.5–21.4) | 20.1  (18.3–22.4) | 20.2  (18.7–21.4) | 0.179^†^ |
|  | Degree of obesity (%) | −0.5  (−7.5–11.0) | −0.9  (−7.5–9.2) | −0.8  (−7.8–7.7) | −1.9  (−9.6–5.0) | 1.2  (−5.7–9.9) | −5.6  (−12.4–2.4) | −3.1  (−7.7–10.4) | 1.9  (−6.1–8.0) | 1.3  (−8.4–11.8) | 2.4  (−5.1–8.6) | 0.127^†^ |
| obesity (+)  n=37 | n | 4 | 6 | 3 | 2 | 8 | 4 | 3 | 2 | 3 | 1 | - |
|  | IR (%) | 25 (0-67) | 33 (0-71) | 100 (100) | 50 (0-100) | 86 (60-100) | 75 (33-100) | 100 (100) | 50 (0-100) | 33 (0-87) | 50 (100) | 0.255^††^ |
|  | HOMA-IR | 2.2  (2.0–4.3) | 2.1  (1.8–3.9) | 6.9 | 4.9 | 4.6  (3.1–5.3) | 3.4  (2.5–7.5) | 2.8 | 6.1 | 2.3 | 2.7 | 0.339^†^ |
|  | BMI (kg/m^2^) | 26.7  (26.4–27.9) | 25.8  (24.6–30.6) | 32.4 | 29.6 | 26.8  (25.1–30.3) | 26.7  (25.8–29.4) | 24.9 | 27.3 | 26.0 | 26.0 | 0.843^†^ |
|  | Degree of obesity (%) | 33.8  (30.9–41.5) | 31.2  (22.8–58.8) | 62.7 | 47.9 | 32.0  (22.9–49.6) | 34.9  (30.6–49.5) | 27.6 | 34.0 | 29.7 | 29.6 | 0.861^†^ |
| overweight (+)  n=70 | n | 12 | 8 | 9 | 6 | 9 | 3 | 8 | 6 | 4 | 5 | - |
|  | IR (%) | 25 (1-50) | 38 (4-71) | 0 | 20 (0-55) | 29 (0-62) | 67 (13-100) | 67 (29-100) | 83 (54-100) | 0 (0) | 0 (0) | <0.009^††^** |
|  | HOMA-IR | 2.1  (1.3–3.4) | 2.1  (1.7–2.8) | 1.6  (0.9–1.9) | 2.1  (1.8–2.6) | 1.7  (1.1–2.6) | 2.9 | 2.8  (2.2–3.1) | 3.6  (2.9–5.6) | 2.0  (1.6–2.3) | 1.3  (1.1–2.2) | <0.006^†^** |
|  | BMI (kg/m^2^) | 22.8  (22.2–23.3) | 22.8  (22.5–23.1) | 22.5  (21.8–23.1) | 22.5  (22.0–23.0) | 22.3  (21.7–23.4) | 22.8 | 22.4  (22.1–23.0) | 23.2  (22.3–23.8) | 22.9  (22.3–23.7) | 22.8  (22.1–23.0) | 0.759^†^ |
|  | Degree of obesity (%) | 13.6  (10.4–16.2) | 13.8  (11.5–15.6) | 12.5  (9.6–15.7) | 14.0  (10.2–16.2) | 14.0  (8.1–15.4) | 13.5 | 10.5  (10.3–15.0) | 15.2  (9.9–16.7) | 16.7  (10.8–21.3) | 12.1  (10.4–15.6) | 0.918^†^ |
| normal  n=335 | n | 41 | 43 | 41 | 38 | 36 | 39 | 24 | 29 | 15 | 26 | - |
|  | IR (%) | 17 (6-29) | 12 (2-21) | 15 (4-26) | 32 (17-48) | 31 (16-46) | 61 (45-76) | 57 (36-77) | 72 (56-89) | 13 (0-31) | 0 (0) | <0.001^††^*** |
|  | HOMA-IR | 1.5  (1.1–2.0) | 1.8  (1.5–2.0) | 1.6  (1.3–2.2) | 2.0  (1.5–2.6) | 1.9  (1.2–2.8) | 2.8  (1.9–3.2) | 2.6  (2.2–3.1) | 2.8  (2.4–3.6) | 1.9  (1.4–2.3) | 1.5  (1.3–1.6) | <0.001^†^*** |
|  | BMI (kg/m^2^) | 19.2  (18.0–20.5) | 19.1  (17.7–19.9) | 19.1  (18.1–20.4) | 18.9  (17.9–20.2) | 19.3  (18.6–20.6) | 18.5  (17.2–19.7) | 19.4  (18.1–19.8) | 19.3  (18.4–20.7) | 19.6  (18.5–20.6) | 19.8  (18.6–20.7) | 0.111^†^ |
|  | Degree of obesity (%) | -3.8  (-8.1–3.0) | -3.6  (-9.3–0.5) | -1.9  (-8.1–2.6) | -3.4  (-10.1–-0.3) | -3.8  (-6.0–2.1) | -9.3  (-12.6–-1.1) | -4.1  (-7.7–2.0) | -1.2  (-7.5–2.5) | -1.4  (-8.3–4.4) | 0.5  (-5.7–4.3) | 0.036^†^* |
| underweight (+)  n=16 | n | 1 | 2 | 3 | 1 | 2 | 1 | 2 | 1 | 3 | 0 | - |
|  | IR (%) | 0 (0) | 50 (0-100) | 0 (0) | 0 (0) | 50 (0-100) | 0 (0) | 50 (0-100) | 100 (100) | 75 (0-88) | - | 0.654^††^ |
|  | HOMA-IR | 1.8 | 2.0 | 0.9 | 2.0 | 2.7 | 2.0 | 2.5 | 2.6 | 2.3 | - | 0.504^†^ |
|  | BMI (kg/m^2^) | 15.2 | 15.0 | 15.3 | 16.0 | 15.7 | 15.1 | 15.9 | 16.3 | 16.0 | - | 0.501^†^ |
|  | Degree of obesity (%) | –23.1 | -26.1 | -20.8 | -16.8 | -21.5 | -25.7 | -19.9 | -16.6 | -19.6 | - | 0.361^†^ |

Data are represented as median (25–75 percentiles [interquartile range]).

IR indicates the prevalence and 95% confidence interval.

HOMA-IR; Homeostasis model assessment of insulin resistance, BMI; body mass index.

†Kruskal-Wallis test. ††Chi-square test. *P < 0.05. **P < 0.01. ***P < 0.001.

**Table S3 | Effect of Benjamini–Hochberg False Discovery Rate Correction on Kruskal–Wallis Test Findings**

| **Index** | **p**  **(unadjusted)** | **tie** | **q**  **(BH-adjusted)** | **Significant**  **(q≤0.05)** |
| --- | --- | --- | --- | --- |
| 1 | 0.103 | 0 | 0.154 | FALSE |
| 2 | 0.032 | 0 | 0.053 | FALSE |
| 3 | 0.009 | 0 | 0.018 | TRUE |
| 4 | 0.007 | 0 | 0.014 | TRUE |
| 5 | 0.001 | 0 | 0.003 | TRUE |
| 6 | 0.001 | 1 | 0.003 | TRUE |
| 7 | 0.001 | 2 | 0.003 | TRUE |
| 8 | 0.001 | 3 | 0.003 | TRUE |
| 9 | 0.001 | 4 | 0.003 | TRUE |
| 10 | 0.563 | 0 | 0.626 | FALSE |
| 11 | 0.257 | 0 | 0.330 | FALSE |
| 12 | 0.137 | 0 | 0.193 | FALSE |
| 13 | 0.049 | 0 | 0.076 | FALSE |
| 14 | 0.013 | 0 | 0.022 | TRUE |
| 15 | 0.001 | 5 | 0.003 | TRUE |
| 16 | 0.001 | 6 | 0.003 | TRUE |
| 17 | 0.001 | 7 | 0.003 | TRUE |
| 18 | 0.570 | 0 | 0.626 | FALSE |
| 19 | 0.293 | 0 | 0.366 | FALSE |
| 20 | 0.147 | 0 | 0.200 | FALSE |
| 21 | 0.049 | 1 | 0.076 | FALSE |
| 22 | 0.001 | 8 | 0.003 | TRUE |
| 23 | 0.001 | 9 | 0.003 | TRUE |
| 24 | 0.001 | 10 | 0.003 | TRUE |
| 25 | 0.540 | 0 | 0.623 | FALSE |
| 26 | 0.358 | 0 | 0.424 | FALSE |
| 27 | 0.157 | 0 | 0.208 | FALSE |
| 28 | 0.001 | 11 | 0.003 | TRUE |
| 29 | 0.001 | 12 | 0.003 | TRUE |
| 30 | 0.001 | 13 | 0.003 | TRUE |
| 31 | 0.886 | 0 | 0.886 | FALSE |
| 32 | 0.614 | 0 | 0.643 | FALSE |
| 33 | 0.011 | 0 | 0.021 | TRUE |
| 34 | 0.005 | 0 | 0.011 | TRUE |
| 35 | 0.001 | 14 | 0.003 | TRUE |
| 36 | 0.669 | 0 | 0.684 | FALSE |
| 37 | 0.004 | 0 | 0.009 | TRUE |
| 38 | 0.002 | 0 | 0.005 | TRUE |
| 39 | 0.001 | 15 | 0.003 | TRUE |
| 40 | 0.012 | 0 | 0.022 | TRUE |
| 41 | 0.005 | 1 | 0.011 | TRUE |
| 42 | 0.001 | 16 | 0.003 | TRUE |
| 43 | 0.614 | 1 | 0.643 | FALSE |
| 44 | 0.115 | 0 | 0.167 | FALSE |
| 45 | 0.333 | 0 | 0.405 | FALSE |
