# Supplementary figures and images for: Reduction of insulin resistance in 14–15-year-old students after the COVID-19 pandemic: a prospective study from Tsunan, Japan
Source: Front Clin Diabetes Healthc. 2026 Jan 7;6:1687294. doi: 10.3389/fcdhc.2025.1687294 (PMC12819293; doi:10.3389/fcdhc.2025.1687294)

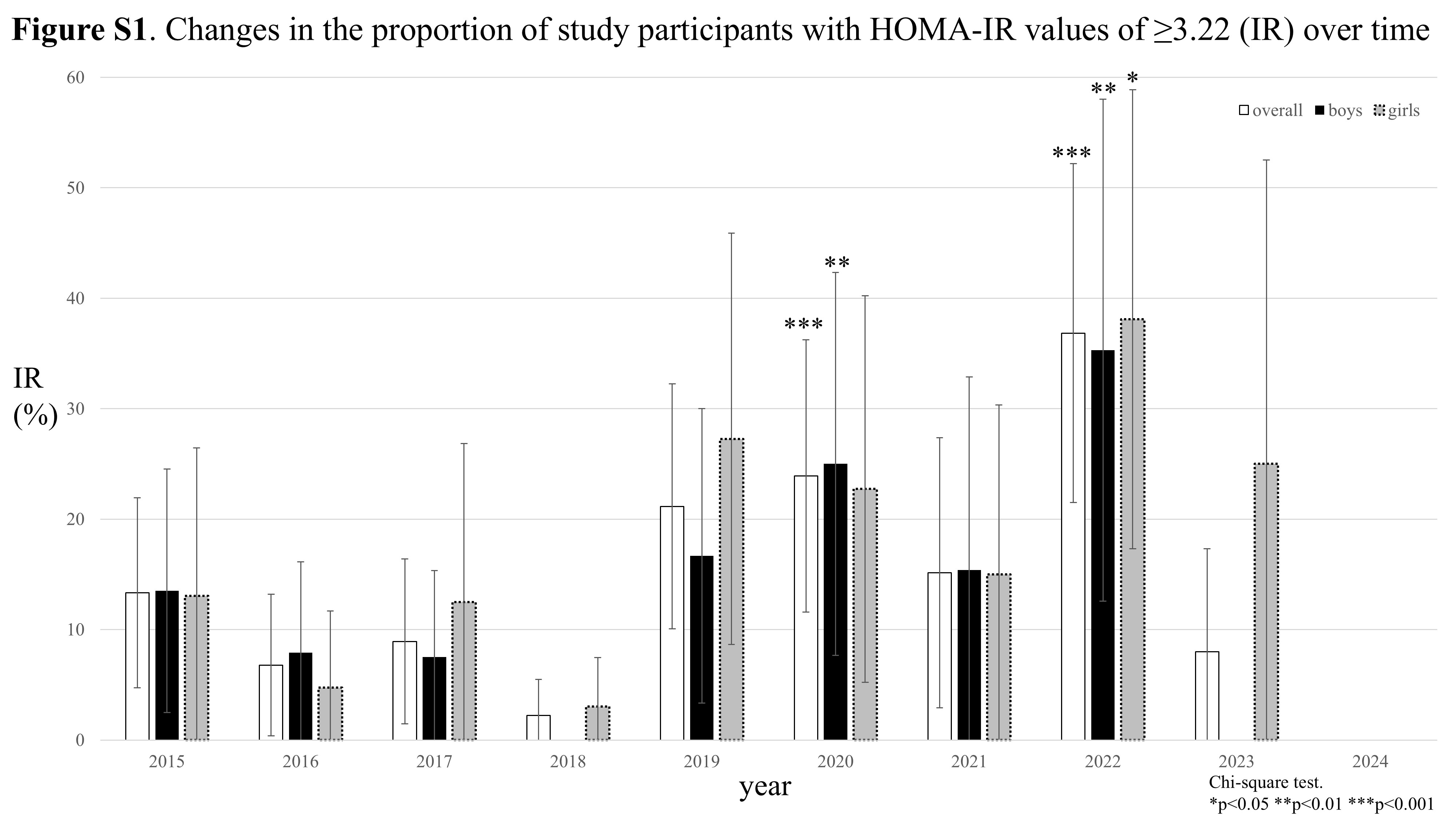

Supplement: Supplementary file 2 [file Image1.jpeg]

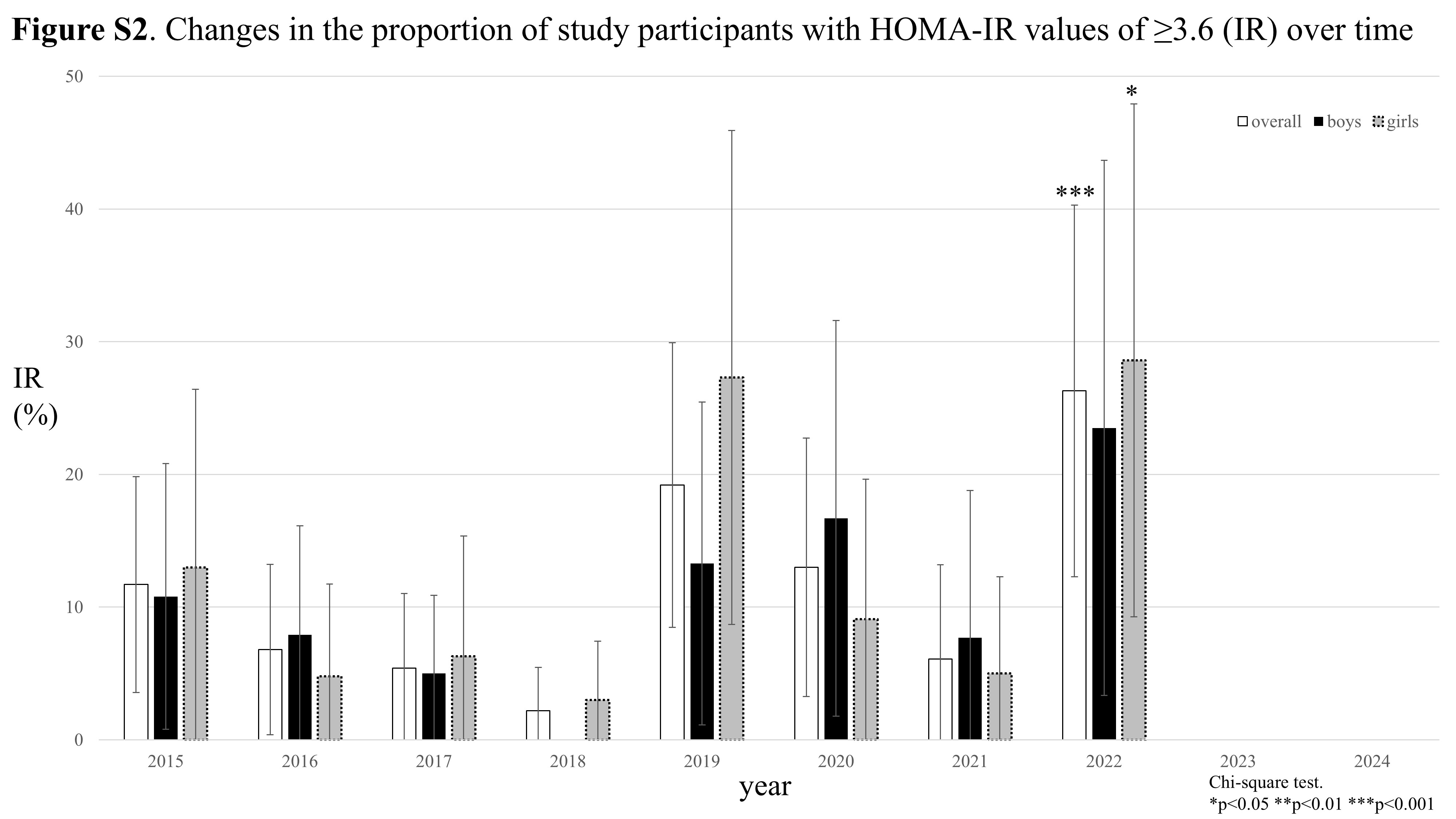

Supplement: Supplementary file 3 [file Image2.jpeg]
